# Morphology and Efficiency of a Specialized Foraging Behavior, Sediment Sifting, in Neotropical Cichlid Fishes

**DOI:** 10.1371/journal.pone.0089832

**Published:** 2014-03-06

**Authors:** Hernán López-Fernández, Jessica Arbour, Stuart Willis, Crystal Watkins, Rodney L. Honeycutt, Kirk O. Winemiller

**Affiliations:** 1 Program in Ecology and Evolutionary Biology, and Department of Wildlife and Fisheries Sciences, Texas A&M University, College Station, Texas, United States of America; 2 Department of Ecology and Evolutionary Biology, University of Toronto, Toronto, Ontario, Canada; University of Windsor, Canada

## Abstract

Understanding of relationships between morphology and ecological performance can help to reveal how natural selection drives biological diversification. We investigate relationships between feeding behavior, foraging performance and morphology within a diverse group of teleost fishes, and examine the extent to which associations can be explained by evolutionary relatedness. Morphological adaptation associated with sediment sifting was examined using a phylogenetic linear discriminant analysis on a set of ecomorphological traits from 27 species of Neotropical cichlids. For most sifting taxa, feeding behavior could be effectively predicted by a linear discriminant function of ecomorphology across multiple clades of sediment sifters, and this pattern could not be explained by shared evolutionary history alone. Additionally, we tested foraging efficiency in seven Neotropical cichlid species, five of which are specialized benthic feeders with differing head morphology. Efficiency was evaluated based on the degree to which invertebrate prey could be retrieved at different depths of sediment. Feeding performance was compared both with respect to feeding mode and species using a phylogenetic ANCOVA, with substrate depth as a covariate. Benthic foraging performance was constant across sediment depths in non-sifters but declined with depth in sifters. The non-sifting *Hypsophrys* used sweeping motions of the body and fins to excavate large pits to uncover prey; this tactic was more efficient for consuming deeply buried invertebrates than observed among sediment sifters. Findings indicate that similar feeding performance among sediment-sifting cichlids extracting invertebrate prey from shallow sediment layers reflects constraints associated with functional morphology and, to a lesser extent, phylogeny.

## Introduction

Adaptive divergence of morphology and behavior has long interested biologists because it provides evidence of biological diversification in response to natural selection. In particular, food intake has an obvious and direct effect on fitness, and as a consequence, foraging behavior has received considerable attention. Modern teleost fishes are particularly good models for comparative research on foraging ecology because the mechanics of their functional morphology are relatively well understood (e.g. [1]–[5]). Studies of fish feeding generally focus on functional morphology and biomechanics of prey capture in the water column (e.g. [6]–[8]), but comparatively little attention has been given to taxa specialized for benthic invertebrate feeding [9], [10].

Consumption of benthic infauna (i.e. prey buried beneath loose sediments, such as sand, silt and particulate detritus) by teleosts usually involves two steps: a) ingestion of a mouthful of sediment using a suction or scooping action, and b) separation of prey items from sediments within the oropharyngeal chamber by processes referred to as sifting [11] or winnowing [9]. The first step involves bringing sediment and buried food items into the mouth cavity. The second step, winnowing of food items from the ingested sediment, involves a series of contractions and expansions of the orobranchial chamber via adduction/abduction of the gill cover and hyoid apparatus. Such action causes cyclical hydraulic currents that move the food/sediment mix back and forth inside the orobranchial chamber. In each cycle, the pharyngeal jaws are used to rake the mix, directing food items into the esophagus and debris towards the gill openings or mouth for expulsion [9].

An ability to extract food particles buried within loose sediments is common among unrelated lineages of teleost fishes that grub or root for buried items [12]. For example, substrate grubbers (rooting with the snout within loose sediments to locate and ingest single food items) include the common carp (*Cyprinus carpio*), callichthyid and doradid catfishes of the Neotropics, and loaches (Cobitidae) of Asia. Digging and sifting (winnowing) behavior is observed among many, if not most of the diverse percomorph fishes; however, there are examples of convergent morphological and behavioral specialists that feed almost exclusively by sifting sediments using the two steps described earlier. These specialized sifters include marine mojarras (Gerreidae), goatfishes (Mullidae), surfperches (Embiotocidae), and certain gobies such as *Awaous* spp. (Gobiidae). Among the Cichlidae, sediment sifting is widespread, with specialized sifters found in African rivers (e.g., *Chromidotilapia* spp., *Tylochromis* spp., *Sargochromis codringtoni*), African lakes (e.g., *Callochromis* spp., *Grammatotria* spp. and *Xenotilapia* spp. in Lake Tanganyika; *Lethrinops* spp. and *Taeniolethrinops* spp. in Lake Malawi) and Neotropical rivers [13], [14]. Among Neotropical cichlids, the South American tribe Geophagini contains two clades with independently derived specialized sediment-sifting genera. The “*Geophagus* clade” includes *Geophagus* sensu lato, *Gymnogeophagus*, *Mikrogeophagus*, and *Biotodoma*, and the “*Satanoperca* clade” includes *Acarichthys*, *Satanoperca*, and *Guianacara*. The Central American heroine genera *Thorichthys* and *Astatheros* are also independently evolved sediment sifters with similar external morphology to that of South American geophagines [13], [15], [16]. Morphological, behavioral and dietary convergence among sediment-sifting Neotropical cichlid clades is widespread. Both clades of sediment-sifting Geophagini and the Heroini *Astatheros* and *Thorichthys* occupy common areas of morphospace (e.g. [13], [15]), share stereotypical sifting-winnowing behaviors [13], [16], and have similar diets with high proportions of benthic items [13], [14], [17]. Among geophagine clades, convergence is also evident in oral jaw biomechanical attributes interpreted as optimized for suction feeding [18]. Additionally, most sediment-sifting taxa within Geophagini have an “epibranchial lobe”, an anteroventral expansion of the first epibranchial bone (e.g. [19]–[21]) that has been found to be correlated with benthic and epibenthic diets [14].

Although many specialized sediment-sifting cichlids appear to have convergent head morphologies (e.g. long snouts, subterminal mouths, [12]–[15]), little is known about the correlation between these morphological attributes and foraging efficiency for benthic prey embedded within sediments as compared to non-sifting taxa. These convergent morphological and behavioral traits may, for example, enable sifters to dig deeper into loose sediments (e.g., longer snouts, eyes positioned high on the head) or winnow with greater efficiency (e.g., large oropharyngeal chamber volume, morphology of gill rakers used in sifting). Alternatively, morphological specialization may not affect foraging depth, but be associated with increased sediment-sifting efficiency by fine-tuning biomechanical attributes associated with winnowing [18] or improving access to shallow-buried prey. We are not aware of studies that have used experimentally manipulated foraging conditions to address foraging behavior and efficiency of sediment-sifting fishes. In this paper, we examine the link between feeding behavior, foraging performance and morphological adaptation to 1) test whether cichlid species sharing a specialized feeding behavior exhibit convergent morphology that is not simply an artifact of evolutionary relatedness (i.e., is adaptive), 2) test whether the morphology and behavior associated with substrate-sifting relates to more efficient performance in terms of foraging for benthic prey than seen in non-sifting taxa lacking these traits.

## Methods

### Ethics statement

This study was performed in accordance with the recommendations in the Guidelines for the Use of Fishes in Research of the American Fisheries Society. The protocol was approved by the Institutional Animal Care and Use Committee of Texas A&M University (AUP# 2005-117). Every effort was made to minimize stress to the fishes used in feeding trials. Morphometrics analyses were performed on specimens on loan from and with permission of the ichthyology collections at the Royal Ontario Museum (ROM), Toronto, Canada, the Museo de Ciencias Naturales de Guanare (MCNG), Guanare, Venezuela, and the Museu de Ciencias da Pontifícia Universidade Catolica do Rio Grande do Sul (MCP), Porto Alegre, Brazil.

### Ecomorphological correlates of feeding

To compare variation in functional attributes associated with sifting and non-sifting foraging tactics, we measured eleven morphological traits of the head of 128 specimens from 27 Neotropical cichlid species including those in our feeding experiments (Table 1, and see below), using specimens requested on loan from the ichthyology collections at the Royal Ontario Museum (ROM), Toronto, Canada, the Museo de Ciencias Naturales de Guanare (MCNG), Guanare, Venezuela, and the Museu de Ciencias da Pontifícia Universidade Catolica do Rio Grande do Sul (MCP), Porto Alegre, Brazil. These species included 13 sediment-sifting species, likely representing two or more origins of sediment-sifting. *Thorichthys ellioti* belongs to a genus of sediment-sifters nested well within the Central American heroines, a clade that includes piscivores, detritivores, rheophilic invertivores, algae eaters, frugivores and generalist feeders (e.g. [13], [17]), while all other sifters examined are South American geophagines. Even within the tribe Geophagini, sifting may have originated more than once; all *Satanoperca* species are more closely related to the non-sifting *Crenicichla* species and to *Guianacara stergiosi* than to any other geophagine sifter, and may be separated from the most recent common ancestor of all geophagine sifters by more than 50 Ma [15], [18]. The morphological dataset also included 14 non-sifting species including piscivores (ex: *Cichla temensis*), detritivores (ex: *Mesonauta egregius*), benthivores (ex: *Dicrossus filamentosus*), generalist feeders (ex: *Guianacara stergiosi* and *Amatitlania siquia*) and a filamentous algae specialist (ex: *Hypsophrys nematopus*) [14]. We measured between 2 and 5 individuals of each species, a sample size previously shown to accurately represent interspecific morphological variation in Neotropical cichlids (e.g. [13]–[15], [22]).

**Table 1:** Species examined in a linear discriminant function analysis of ecomorphology of sediment-sifting and non-sifting cichlids.

Sediment-sifters	Non-sifters
*Satanoperca mapiritensis* (G)	*Guianacara stergiosi* (G)
Satanoperca daemon* (G)	*Crenicichla sp. “orinoco lugubris”* (G)
*Geophagus' brasiliensis* (G)	*Crenicichla sp. “orinoco wallaci”* (G)
*Geophagus abalios* (G)	*Crenicichla sveni* (G)
*Geophagus dicrozoster* (G)	*Crenicichla geayi* (G)
Geophagus brachybranchus*(G)	*Dicrossus filamentosus* (G)
Geophagus' steindachneri* (G)	*Hoplarchus psittacus* (H)
*Gymnogeophagus rhabdotus* (G)	Amatitlania siquia* (H)
*Gymnogeophagus balzanii* (G)	Hypsophrys nematopus* (H)
*Biotodoma wavrini* (G)	*Mesonauta egregius* (H)
*Mikrogeophagus ramirezi* (G)	*Cichlasoma orinocense* (Cs)
Mikrogeophagus altispinosus* (G)	*Cichla temensis* (Ci)
Thorichthys ellioti* (H)	*Cichla orinocensis* (Ci)
	*Astronotus sp.* (A)

Letters in parenthesis identify the Neotropical cichlid tribes included in the morphological analysis: Geophagini (G), Heroini (H), Cichlasomatini (Cs), Cichlini (Ci) and Astronotini (A). Species used in feeding experiments are highlighted with an asterisk.

In addition to recording SL (distance between the tip of the upper lip with mouth completely closed to the midpoint of the caudal peduncle where the caudal fin rays insert into the hypural plates), various head measurements were taken with vernier calipers to the nearest millimeter. Measurements of pharyngeal attributes were performed after dissection of the pharyngeal basket using an ocular micrometer attached to a dissecting stereomicroscope to the nearest tenth of a millimeter. Measurements taken are as follows (abbreviations in parentheses refer to illustrations of measurements in Fig. S1): *head length* (HL) measured from the tip of the upper lip with the mouth completely closed to the caudal edge of the operculum; *head height* (HH) as the vertical distance through the center of the eye between the dorsal and ventral edges of the head; *gape width* (GW) as the horizontal internal distance between the tips of the premaxilla with the mouth fully open and protruded; *eye position* (EP) as the vertical distance between the center of the eye and the ventral edge of the head; *eye diameter* (ED) as the longest horizontal distance between the anterior and posterior edges of the eye; *snout length* (SnL) as the distance from the center of the eye to the center of the upper lip (i.e. the symphysis of the premaxilla) with mouth closed; *ceratobranchial length* (CbL) measured as the straight distance between the joint of the basibranchial with the first ceratobranchial arch and the joint between the first ceratobranchial and the epibranchial; *ceratobranchial gill-raker space* (CbGRsp) as the average distance between gill rakers on the first ceratobranchial arch from five measurements; *epibranchial lobe length* (EBL) the longest distance between the base of the epibranchial lobe (if present) and its tip, excluding gill rakers; *lower pharyngeal jaw width* (LPJW) measured as the maximum external distance between the horns; and *lower pharyngeal jaw length* (LPJL) as the maximum distance from the imaginary midline between the caudal edge of the horns and the anterior-most tip of the plate.

With the exception of epibranchial lobe length (which was expressed as a proportion of head length to accommodate values of 0), a phylogenetically-corrected least-squares linear regression was performed to account for variation in morphological traits resulting from body size variation. All species were analyzed in a single regression of each morphological variable against SL, and the residuals of these phylogenetically-corrected regressions were used as size-corrected character values. A phylogenetically-corrected least squares regression includes a transformation by a variance-covariance matrix derived from phylogenetic branch lengths [23]. This transformation accounts for the fact that species trait values are not independent of one another as a result of shared evolutionary history, which would otherwise violate an assumption of regression analysis [23]. We used a modified version of the “phyl.resid” function from the “phytools” R package [23] to allow for multiple individuals of the sample species which is described below (see File S1 for R script, function “phyl.resid.intra”). Morphological variables, including body size (SL), were log-transformed prior to regression analysis, to account for skew associated with body size dependent traits.

We adjusted the C matrix (evolutionary variance-covariance matrix) which summarizes the shared evolutionary history between species pairs [24] such that an individual within a species shares equal evolutionary history with all other members of that species. An example of the C matrix and modified C matrix is given below for 3 species, each of which has 2 individuals in matrix C_n_. Although, realistically, members of different populations or sub-species may not share equal evolutionary history, we feel this is a reasonable assumption given the evolutionary time-scales being considered in these analyses and the fact that all tested fishes are full to half siblings (see [15]). We also found that the mean of the residuals calculated using C_n_ were identical to the residuals calculated using C and the mean species character values and therefore C_n_ and C produce consistent results at least at the species level.

v = total length of tree

c_i,j_ = shared evolutionary history (expected covariance) of species i and j
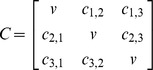


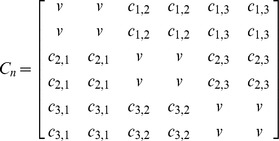



**Figure 1:**
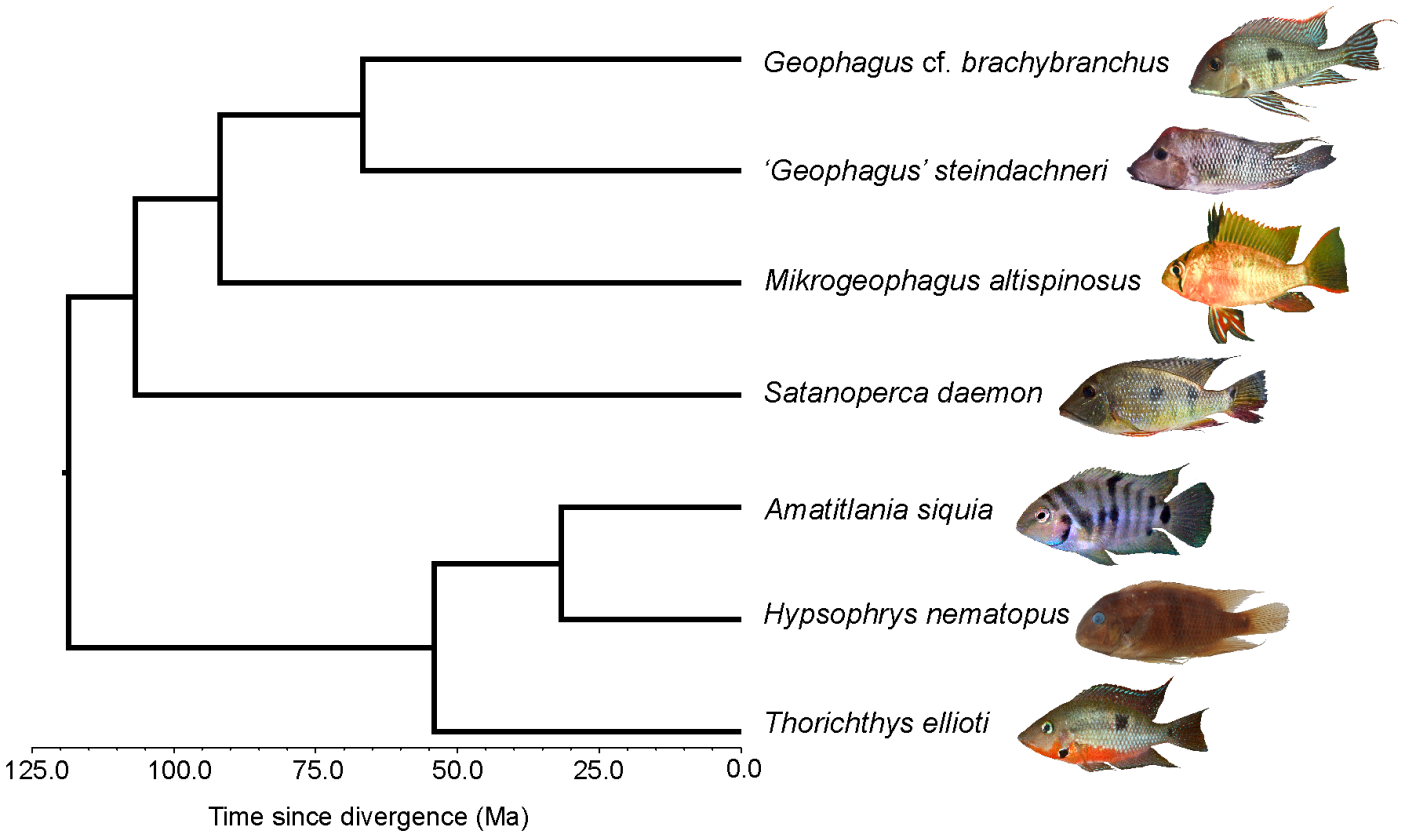
Species of Neotropical cichlids used in foraging experiments. A *Geophagus* cf. *brachybranchus*, B ‘*Geophagus*’ *steindachneri*, C *Mikrogeophagus altispinosus*, D *Satanoperca daemon*, E *Thorichthys ellioti* (picture shown is of the congeneric *T.* cf *meeki*), F *Amatitlania siquia*, G *Hypsophrys nematopus*. Phylogeny and times of divergence follow López-Fernández et al. [15].

The residuals of the regression of each variable on SL were used in a linear discriminant analysis (LDA) comparing the sifter and non-sifter classes. Assignment of individuals of each species to each of the two classes was based on Winemiller et al. [13], Hulsey and de León [16], and López-Fernández et al. [14], [15]. We used a procedure similar to that of a phylogenetic ANOVA [25] to determine whether the results of the LDA could have occurred under a random-walk, Brownian motion process or whether an adaptive process is more likely. Phylogenetic correction was based on the Neotropical cichlid maximum clade credibility (MCC) chronogram provided by López-Fernández et al. [15] after pruning it to include the species used in this study (Fig. 1). Following phylogenetic ANOVA [25], a null distribution of F values for the LDA were generated from data produced from 1000 BM simulations based on this tree; observed F-values were compared against this simulated distribution. The p-value summarizes the frequency of BM simulations that produced a higher F statistic than the observed data. To account for intraspecific variation, we sampled 24 new observations for each species based on its simulated mean value and its observed standard deviation in feeding performance (“rnorm” from R package “stats”). We also calculated how frequently the discriminant function correctly classified sifters vs. non-sifters from the BM simulated datasets and compared this to the observed results. See File S2 for phylogenetic LDA R script (function “phyl.lda”).

### Species included in live experimental trials

Of the 27 species examined in the morphological analysis, we selected five representative species of sediment-sifting Neotropical cichlids and two non-sifting species to examine foraging efficiency. Four of these species (*Geophagus* cf. *brachybranchus*, *‘Geophagus’ steindachneri*, *Mikrogeophagus altispinosus* and *Satanoperca daemon*) belong to two potentially convergent clades within the South American tribe Geophagini [26] while the fifth species, *Thorichthys ellioti*, is part of a specialized sediment-sifting genus in the Central American tribe Heroini, a group that lacks an epibranchial lobe but displays morphological and dietary attributes convergent with those of sediment sifters in the tribe Geophagini [13], [15], [16]. All of these sediment-sifting species inhabit river and stream habitats with sand, mud, particulate organic matter or a combination of these sediments (e.g. [13], [15]–[17]). Two species with different morphology from that of sediment-sifters, and therefore not expected to perform well when feeding on benthic invertebrates, were the Central American heroine cichlids *Amatitlania siquia* (a morphologically generalized omnivore) and *Hypsophrys nematopus*, a filamentous benthic algae specialist [13], [15].

### Experimental setup

We used an experimental protocol to estimate efficiency of fishes feeding on invertebrates buried beneath layers of sand at variable depths. Fish used in the experiments belonged to cohorts produced in our laboratory from parental stocks obtained from the pet-trade and raised together in the same aquarium room where experiments were performed. Water in all aquariums was prepared with de-ionized water remineralized with a salt mixture (2 parts CaCO_3_, 2 parts MgCl_2_, 1 part CaCl_2_, and 1 part MgSO_4_ by volume, for a final conductivity of <50 uS; ∼1 tbsp/210 L). Commercially available frozen chironomid larvae (Hikari brand bloodworms) were used in all experiments. Chironomid larvae (Diptera) are an important dietary component of many benthivorous Neotropical cichlids [13], [14]. Frozen chironomid larvae were thawed by gently rinsing them with warm tap water and then floating them in a 30% solution of sucrose. This procedure allowed for undamaged larvae to be recovered with a fine mesh net as they floated on the top of the solution, while damaged exoskeletons and other debris sank to the bottom [27]. Once recovered, whole larvae were rinsed with tap water to eliminate the sucrose, and gently blotted with a paper towel until moist but without water visible on the material. An electronic balance was used to partition larvae into 5-g portions. These portions were either used immediately or frozen for later use. To ensure that no weight was lost during freezing, thawed portions were weighed again prior to use in trials.

For each trial, weighed chironomid larvae were evenly spread across the bottom of a “20-gallon-long” (75.7 L, bottom area = 2,250 cm^2^), all-glass aquarium. Clean pool-filter sand of uniform grain-diameter was either left bare (0 cm substrate depth) or carefully spread over the chironomid larvae at a uniform depth (1, 2 or 3 cm). Freshly prepared water (see above) was added to the tank without disturbing the sand (a plastic tray was temporarily placed over the sand during filling and gently removed afterwards). During trials, an airstone provided aeration. Both holding and experimental tanks were maintained in the aquarium room at a temperature between 26–28°C.

Before any data collection, we performed a series of trial experiments to determine the suitable amount of food and trial duration that would allow discrimination of performance among individuals and species. In experimental trials, fish were offered a known amount of food and allowed to forage for a fixed period of time. After each trial, the difference between the initial amount of food and the amount remaining in the experimental tank was used as an indicator of feeding efficiency (see below). By combining different initial amounts of food and different foraging periods, we determined that an initial amount of 5 g of food (approximately ∼0.002 g/cm^2^ or an average of ∼730 individual chironomids) and a trial duration of 3 h consistently yielded measurable amounts of uneaten food. Preliminary experiments were run with aquarium-reared *Geophagus* cf. *brachybranchus* (*N* = 6, 40–65 mm standard length, SL) and *Mikrogeophagus altispinosus* (*N* = 6, 35–50 mm SL). We then performed a series of control tests without any fish (*N* = 10) to determine the mean and variance of food weight loss associated with handling and other aspects of the experimental procedure.

Feeding experiments were started within 20 min after the experimental tanks had been set up. A single fish that had not eaten for 24 h was introduced into each experimental tank and permitted to forage for 3 h, during which time the aquarium room was not disturbed. At the end of each trial, the fish was removed, measured for standard length (SL), and placed in a stock tank that identified individuals that had been tested. To avoid bias associated with individual subjects, each fish was used in a single feeding trial. After each trial, the sand and uneaten chironomid larvae were removed from the tank, and placed in a container with a 30% sucrose solution. The sand was gently stirred until all chironomid larvae had been recovered after floating to the surface of the solution (see above). Chironomid larvae were then rinsed, blotted dry and weighed as described above. Six experimental replicates at four substrate depths (no sand, 1, 2 and 3 cm) were performed for each of the seven cichlid species, so that a total of 24 individuals of each species were tested: *Geophagus* cf. *brachybranchus* (45–69 mm SL), *Mikrogeophagus altispinosus* (35–50 mm SL), ‘*Geophagus’ steindachneri* (45–63 mm SL), *Satanoperca daemon* (53–62 mm SL), *Thorichthys ellioti* (35–66 mm SL), *Amatitlania siquia* (40–66 mm SL), and *Hypsophrys nematopus* (45–60 mm SL).

At the conclusion of the experiment, it was obvious that one species, *Hypsophrys nematopus*, used a different foraging tactic to extract chironomid larvae buried under sand. The other six cichlid species repeatedly thrust their jaws into the loose sediments to obtain sand mixed with food, and then winnowed the food from the sand within the confines of the oropharyngeal chamber. This action typically was performed at frequent intervals (every 3–10 sec) at positions throughout the aquarium. In contrast, *Hypsophrys* used its mouth as well as sweeping movements of its body and fins to excavate large pits in the sand from which it consumed exposed food items one at a time without ingesting sand. Therefore, we designed a second experiment to test the hypothesis that this foraging tactic increases feeding efficiency when buried food is patchily distributed rather than evenly dispersed. The protocol of the second experiment was the same as the first, except that the 5 g of chironomid larvae were placed on the bottom of the tank in two equal clumps before the bottom of the tank was carefully covered with a layer of sand. We tested the clumped food pattern at 0, 1, 2 and 3 cm depths of sand using a different individual *Hypsophrys* for each trial. Six replicate trials at each depth were run using individuals from the same cohort (each used only once) for each of the 4 treatments.

### Measure of feeding efficiency

Feeding efficiency was quantified as the difference between the initial wet weight and the recovered wet weight of chironomid larvae consumed by each experimental fish. To account for differences in body size among individual fish, the amount of larvae consumed was standardized per unit of consumer body length (ln[SL in mm]). Statistical significance of feeding behavior (sifter, non-sifter) or species and their interaction was evaluated as predictors of feeding performance using phylogenetically corrected analysis of covariance (ANCOVA) with sand-depth as the covariate. Phylogenetic ANCOVA was performed following Garland et al. [25], which tests whether the results of an ANCOVA could have been generated under a process of Brownian motion (BM) evolution (i.e., a neutral, random walk). We used a modified version of the function “phylANOVA” (R package “phytools”, [23]) and the function “ancova” (R package “HH”) to carry out these analyses. The R code for these modified functions can be found in File S3 (function “phylANOVA.intra”). In the case of *Hyposphrys*, a factorial (2×4) analysis of variance based on untransformed data was used to test for statistical differences between dispersion patterns (e.g. clumped versus evenly distributed chironomids) and sand depths.

## Results

### Ecomorphology

**Figure 2:**
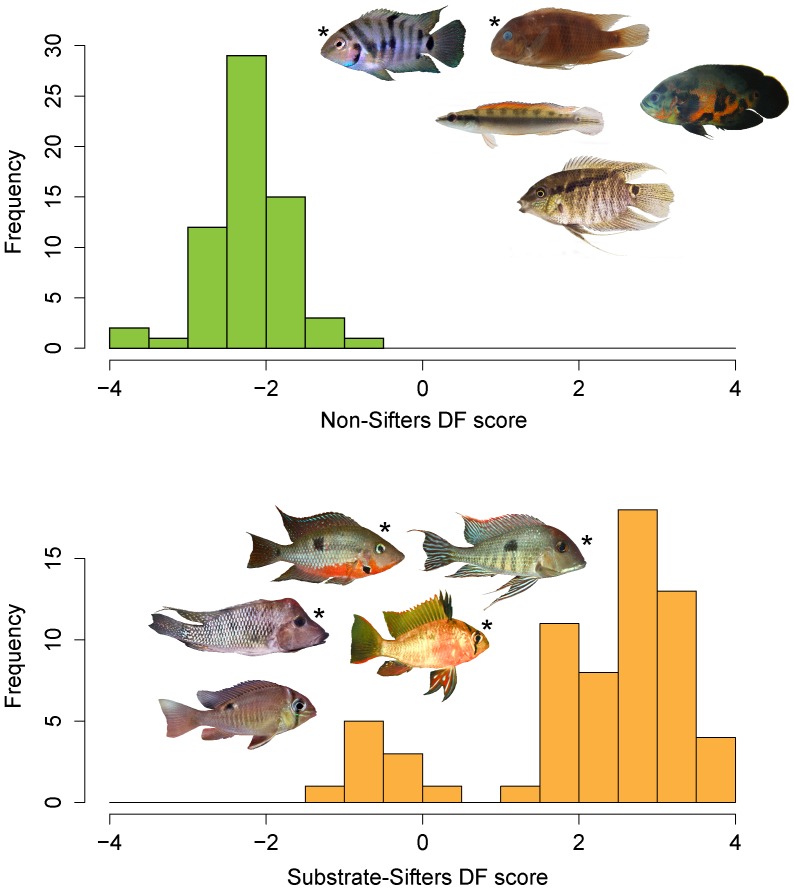
Linear discriminant function analysis (LDA) of morphological attributes in 27 species of Neotropical cichlids. LDA produced an axis of variation that effectively separated non-sifters (top panel) from specialized sediment-sifting species (bottom panel) by their morphological attributes. Among sediment-sifters, the model distribution to the left represents individuals “misclassified” by the LDA analysis as non-sifters, including *Thorichthys ellioti* (5/5), *Mikrogeophagus ramirezi* (4/4) and *Biotodoma wavrini* (1/5). Images marked with an “*” depict genera used in feeding efficiency experiments.

Analysis of two classes of feeding behavior resulted in one discriminant function of morphology (i.e. number of classes - 1) that strongly separated specialized sifters from non-sifters. In general, sifters tended to have larger eyes placed more dorsally, wider gapes and pharyngeal jaws and deeper heads than non-sifting species. The presence of the epibranchial lobe was also characteristic of sifters in species from both clades of Geophagini, compared with non-sifting geophagines and all heroines, both of which lack the lobe (Table 2). The discriminant function of the observed data was able to accurately predict feeding behavior from the residuals of the morphological characters for 93% of individuals examined (Fig. 2). Those specimens that were identified as belonging to the wrong class (sifter or non-sifter) were either *Thorichthys ellioti (5/5), Biotodoma wavrini (1/5)* or *Mikrogeophagus ramirezi (3/4)*, and all of these were sediment sifters misclassified as non-sifters. Linear discriminant analysis was better able to explain observed variation in morphological traits of the two classes compared to BM expectations, based on the null (simulated) distribution of F-values (p<0.001). Furthermore, the linear discriminant functions of the BM simulated datasets were equally as accurate or more accurate at identifying sifters and non-sifters in only 2.7% of simulations. Morphological convergence among sediment-sifters within Geophagini (e.g. *Satanoperca* and *Geophagus*) and at least the heroine genus *Thorichthys* is, therefore, unlikely to have arisen by chance under a BM evolutionary process.

**Table 2:** Coefficients of the linear discriminant function of ecomorphology for each variable examined.

	Coefficients	Non-sifter means	Sediment-sifter means
Head length	1.34	−0.00855(0.0403)	0.000114(0.0273)
Head height	3.80	−0.0539(0.183)	0.0831(0.0381)
Gape width	5.51	−0.010465(0.0863)	0.0165(0.0847)
Eye position	−5.18	−0.0488(0.171)	0.0739(0.0465)
Eye diameter	7.27	−0.0283(0.0794)	0.0391(0.0385)
Snout length	0.358	−0.0265(0.0801)	0.0576(0.0455)
Ceratobranchial length	−8.77	−0.0234(0.0460)	0.0103(0.0509)
Ceratobranchial inter gill raker spacing	0.313	0.0193(0.0566)	−0.0482(0.117)
Epibranchial lobe length	8.21	0(0)	0.433(0.166)
Lower pharyngeal jaw width	−7.20	0.0296(0.0890)	−0.0373(0.0476)
Lower pharyngeal jaw length	3.88	−0.0151(0.0572)	0.00109(0.0462)

Mean values for each variable for sediment sifters and non-sifters (standard deviations in parentheses).

### Feeding efficiency

**Figure 3:**
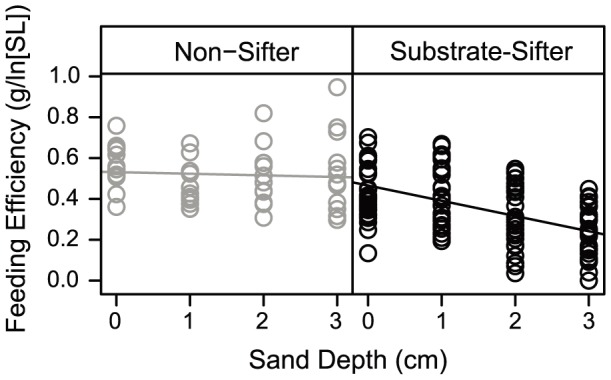
Mean consumption of chironomid larvae buried at 0, 1, 2, or 3-sifting (2 species) and sediment-sifting (5) Neotropical cichlids. Consumption by each species is illustrated in S2.

Phylogeny-corrected ANCOVA showed a significant difference in mean feeding performance between sediment-sifting and non-sifting taxa (F_1,164_ = 48.46, p<0.0001), and feeding performance varied significantly with sand depth as a covariate (F_1,164_ = 33.38, p<0.0001). There was a significant interaction between feeding behavior and sand depth (F_1,164_ = 9.81, p<0.01), with sediment sifters having a significantly more negative relationship between feeding performance and depth (Fig. 3). We observed that the difference in mean feeding performance (between sifters and non-sifters) could have occurred by chance under a Brownian motion process (p = 0.069), but both the effect of depth on feeding performance and the interaction between feeding behavior and sand depth differed significantly from that generated under a random walk, BM evolutionary process (both p = 0.001). The difference in the relationship between feeding performance and depth in sediment-sifters (decrease in performance with depth) versus non-sifters (performance roughly equal across depths) is therefore unlikely to have occurred simply as an artifact of shared evolutionary history among the taxa examined.

Species exhibited significantly different mean feeding performance under a phylogeny-corrected ANCOVA (F_6,154_ = 17.5, p<0.0001). Feeding performance varied significantly with sand depth (F_1,154_ = 42.2, p<0.0001; S2), and there was a significant interaction between species and sand depth on feeding performance (F_6,154_ = 3.62, p<0.01). However, the difference in mean feeding performance between species could have occurred under BM evolution (p = 0.218). While sand depth still represented a significant covariate compared to BM evolution expectations (p = 0.001), the interaction of sand depth and species on feeding performance was marginally non-significant (p = 0.067) compared to BM expectations. Therefore, changes in feeding performance with depth were more strongly associated with feeding behavior (which was significantly different from BM expectations) than with taxonomy (which was not significantly different from BM expectations).

**Figure 4:**
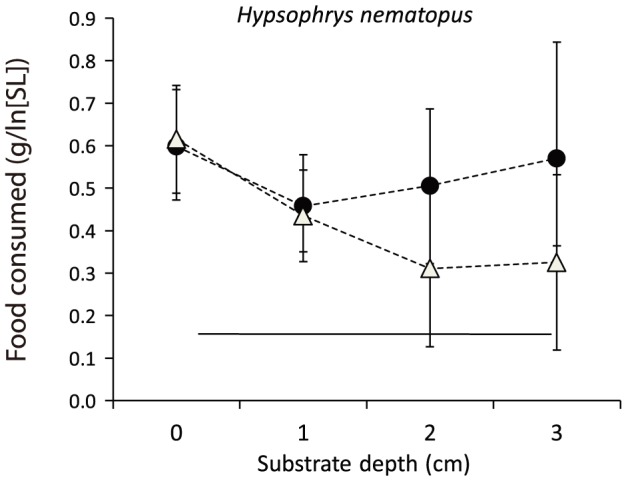
Mean consumption of chironomid larvae buried at 0, 1, 2, or 3 cm depth by the heroine Neotropical cichlid *Hypsophrys nematopus*. Even (filled circles) versus clumped (empty triangles) distributions. The horizontal line indicates weight loss of chironomid larvae in control tanks.

Mean foraging efficiency declined with increasing sand depth for sediment-sifting species (Fig. 3, S2). *Amatitlania siquia* and *Hypsophrys nematopus* revealed small differences in mean foraging efficiency in relation to sand depth, with no overall trend, and the standard deviation of mean foraging efficiency increased with sand depth for *Hypsophrys* (Fig. 4). This unusual pattern for *Hypsophrys* was associated with a foraging strategy that was unique among species tested. *Hypsophrys* dug large pits using its mouth to move sand by grasping or suctioning, followed by ejection of the particles; and also by performing sweeping movements with its body and fins to excavate large pits in the sand. Chironomid larvae exposed within a pit were consumed individually without any obvious ingestion of sand. A second experiment tested the effect of clumped versus even-dispersed chironomid larvae on the foraging efficiency of *Hypsophrys*: there was a significant effect of dispersion pattern (*F*_1,40_ = 4.89, *p*<0.033) and sand depth (*F*_3,40_ = 3.07, *p*<0.039), and their interaction was non-significant (*F*_3,40_ = 1.61, *p*<0.202). The mean foraging efficiency was nearly the same for clumped and evenly dispersed chironomid larvae at 0 and 1 cm sand depth, but mean foraging efficiency was greater for evenly-dispersed food items at 2 and 3 cm (Fig. 4, circles).

## Discussion

Based on a linear discriminant analysis of 27 species of Neotropical cichlids, morphological convergence among sifters (vs. non-sifters) was greater than expected by chance under a Brownian motion evolutionary process (we obtained a higher correct classification frequency in only 2.7% of BM simulations). The discriminant function was not 100% effective at predicting feeding mode from ecomorphology, which may relate to an inherent property of morphological and mechanical diversity. The principle of “many-to-one mapping” of form and function allows taxa to converge in functional output with different morphological adaptations, and can weaken the relationship between morphological adaptation and ecological performance [28]–[31]. It is possible that because many functional systems are incorporated into sediment sifting (ex: suction feeding ability, hyoid depression, pharyngeal jaw movement, oral jaw protrusion), this principle may have resulted in functionally equivalent morphological variation with respect to sediment-sifting performance. Under “many-to-one-mapping”, ancestral trait values form the starting point for potentially differing morphological evolutionary trajectories that nevertheless result in functionally equivalent endpoints [29], [30], [32]. *Thorichthys ellioti* specimens may be functionally but not morphologically convergent with geophagine substrate sifters simply as a result of different evolutionary starting points (Fig. 2). More functionally informative traits (e.g. lever biomechanics, jaw protrusion) may have a greater potential to demonstrate morphological convergence among feeding strategies in future studies. Given the possibility of functional redundancy, the strength of convergence observed in ecomorphological traits among sifters versus non-sifters was somewhat surprising, and supports a role for adaptive constraint on morphological diversification associated with this specialized feeding behavior in Neotropical cichlids.

The feeding efficiency experiments did not reveal a foraging advantage for sediment-sifting Neotropical cichlids when feeding on small benthic invertebrates buried in increasingly deep sand. Instead, the specialized sediment-sifting geophagines and the heroin *Thorichthys* all showed a sharp decline in their ability to capture buried prey as substrate depth increased (Figs. 3, S2). *Hypsophrys nematopus*, a Central American heroine with a relatively small head and small compact jaws ill-suited for scooping and sifting sediments, displayed high foraging efficiency at all sand depths. *Amatitlania siquia*, another Central American heroine, has a generalized cichlid morphology and also revealed relatively high foraging efficiency (S2). By revealing that specialized sifters are not more proficient in extracting chironomid larvae buried in sand, our findings suggest that the distinct morphological attributes of sediment-sifting cichlids do not provide an advantage for digging deeper into loose sediments in search for prey. Rather, the negative relationship between feeding performance and sand depth suggests that sediment-sifting taxa forage most efficiently for prey embedded in sediments at shallow depths.

Sediment-sifting among cichlids is a specialized behavior that apparently evolved independently among phylogenetically disparate taxa possessing similar but not necessarily identical morphological traits [14], [15], [18]. Sifting allows fish to process large amounts of sediment efficiently. Our results indicate that deeply buried food items are less accessible for the sifters we tested. Interestingly, this specialized morphology and behavior for sorting food from sediments does not appear to result in a high degree of dietary specialization, at least with regards to prey types. López-Fernández et al. [14] reported that sediment-sifting geophagine cichlids feed on diverse benthic/epibenthic invertebrates and detritus. Bastos et al. [33] found that gastropods and vascular plant fragments were the most common items among stomach contents of ‘*Geophagus’ brasiliensis*, and Winemiller et al. [13] found the diet of *Geophagus* spp. to be composed predominantly of insects, seeds/fruit and detritus. All these resources are available to fishes at the interface between the water column and the shallow horizons of sandy or muddy substrates. Sediment sifting could, nonetheless, facilitate resource partitioning in terms of differential efficiencies for sediment types within different habitats and microhabitats (and see [17], [34], [35]).

Among Neotropical cichlids, sediment sifters have comparatively high species richness. Typical lowland South American communities can include a large number of coexisting sediment-sifting taxa. For example, communities in the Cinaruco River (Orinoco Basin) and Casiquiare River (Amazon Basin) in Venezuela harbor coexisting species of *Geophagus*, *Satanoperca*, *Biotodoma*, *Apistogramma* and *Biotoecus* [34], [36], [37], all of which have large components of benthic or epibenthic invertebrates in their diet [14]. Although not as diverse as South American Geophagini, Central American Heroini contains several sediment-sifting species. Soria-Barreto and Rodíles-Hernández [35] reported two species of sediment-sifting *Thorichthys* syntopic within the Usumacinta River Basin in Mexico. In the Bladen River of Belize, *Thorichthys meeki* coexists with sediment-sifting *Astatheros robertsoni* [17]. In natural habitats, cichlids forage on a variety of substrates, including sand, silt, and fine and coarse particulate organic matter. The ability to thrust the jaws deep into the substrate may not be as important as being able to separate small prey from sediments of different types and sizes. In most habitats, meiofauna density is probably greatest at shallow substrate depths, and selection favoring deeper thrusts may not be strong for benthivorous cichlids. Dietary segregation among sifters could be facilitated by interspecific differences in biomechanical attributes [18]. For example, species with relatively small mouths and short snouts, such as *Biotodoma wavrini* and *Mikrogeophagus ramirezi*, may be better able to pick and then sift benthic invertebrates from the surface of sediments. Species with larger gapes, such as *Satanoperca* spp., *Geophagus* spp. and *Retroculus lapidifer* [14], may be more efficient winnowers of invertebrates embedded within sediments. We did not examine the role of prey size, and the chironomid larvae used in our experiments may have been too large to reveal a foraging advantage for *Thorichthys* and the geophagine species. To test the hypothesis that geophagines and morphologically and behaviorally convergent heroine cichlids, such as *Thorichthys* and *Astatheros* species, are more efficient foragers for tiny invertebrate components of the infauna, future experiments should manipulate prey size and sediment particle size.

An unexpected finding from our experiments was the divergent foraging tactic displayed by *Hypsophrys nematopus*. This species was included in the study because it has a morphology that is poorly suited for effective scooping and sifting of sediments, and as a result, was expected to provide a sort of null case for comparison with sediment-sifting species. However, *Hypsophrys* was able to consume buried chironomid larvae efficiently by moving large amounts of sand with the mouth as well as by sweeping motions of the body and fins. Species of the genus *Hypsophrys* inhabit streams and rivers with moderate to fast flow velocities, and excavate holes for nesting and brood guarding (Coleman, 1999). While not as specialized for excavation of holes for nesting, *Amatitlania siquia*, the popular convict cichlid of the aquarium hobby, is well known for its habit of moving large amounts of loose sediment to construct nests. Thus, these Central American species appear to have behavioral repertoires adaptive for nesting as well as locating invertebrate prey buried in sand or other loose sediments.

Functional morphology of feeding in cichlids and other fishes has been studied extensively, but most investigations have focused on use of the oral jaws to capture and manipulate elusive prey. It should be recognized that a significant portion of the family Cichlidae, as well as the global diversity of fishes, consists of species that sift food items from sediments via winnowing within the orobranchial chamber. Our experiments revealed aspects of morphology that may influence feeding efficiency among sediment-sifting cichlids and may influence feeding efficiency in other substrate sifting fishes. Our results show a direct impact of feeding behavior specialization on ecological performance and a corresponding convergence in morphological traits, both of which could not be explained by random-walk evolutionary processes. These results also included some unexpected correlations between morphology and feeding that further illustrate the complexity of relationships between morphology, behavior, and ecology. Given the commonness of sediment sifting within the Cichlidae, further research that integrates functional morphology and ecological performance for this foraging mode should enhance our understanding of evolutionary diversification in this hyperdiverse fish family. Studies in cichlids may also contribute to understand one of the most widespread behaviors in teleost fishes.

## Supporting Information

Figure S1 **Illustration of morphometric measurements used in this paper.** A. Body and head measurements. B. Lower pharyngeal jaw measurements. C. First gill arch measurements. All measurements as linear distances between points. Abbreviations follow those given in “Methods” section. See text for descriptions.
(EPS)

Figure S2 **Mean consumption of chironomid larvae buried at 0, 1, 2, or 3 cm depth by seven species of Neotropical cichlid fishes.**
(EPS)

File S1 **R code for function “phyl.resid.intra” which carries out a phylogenetic size correction for more than one individual per species/tip.** See methods and supplementary file for details.
(R) Click here for additional data file.

File S2 **R code for function “phyl.lda” which carries a linear discriminant analysis and compares the results to a set of Brownian Motion simulated values.** The function can include more than one individual per species/tip. See methods and supplementary file for details.
(R) Click here for additional data file.

File S3 **R code for function “phylANOVA.intra” which performs and ANOVA/ANCOVA analysis by comparing the results to a set of Brownian Motion simulated values.** The function can include more than one individual per species/tip. See methods and supplementary file for details.
(R) Click here for additional data file.
